# Increased DJ-1 expression under oxidative stress and in Alzheimer's disease brains

**DOI:** 10.1186/1750-1326-4-12

**Published:** 2009-02-25

**Authors:** Stéphanie Baulac, Hope Lu, Jennifer Strahle, Ting Yang, Matthew S Goldberg, Jie Shen, Michael G Schlossmacher, Cynthia A Lemere, Qun Lu, Weiming Xia

**Affiliations:** 1 Center for Neurologic Diseases, Department of Neurology, Brigham and Women's Hospital, Harvard Medical School, Harvard University, Boston, MA 02115, USA; 2 Harriet and John Wooten Laboratory of Alzheimer's Disease Research, The Brody School of Medicine, East Carolina University, Greenville, NC 27834, USA; 3 Department of Anatomy and Cell Biology, The Brody School of Medicine, East Carolina University, Greenville, NC 27834, USA; 4 Departments of Neurology and Psychiatry, The University of Texas Southwestern Medical Center, Dallas, TX 75390-8813, USA

## Abstract

Mutations in the DJ-1 gene have been linked to autosomal recessive familial Parkinson's disease. To understand the function of DJ-1, we determined the DJ-1 expression in both zebrafish and post mortem human brains. We found that DJ-1 was expressed early during zebrafish development and throughout adulthood. Knock down (KD) of DJ-1 by injection of morpholino did not cause dramatic morphologic alterations during development, and no loss of dopaminergic neurons was observed in embryos lacking DJ-1. However, DJ-1 KD embryos were more susceptible to programmed cell death. While a slight reduction in staining for islet-1 positive neurons was observed in both DJ-1 KD and H_2_O_2 _treated embryos, the number of apoptotic cells was significantly increased in both KD and H_2_O_2 _treated embryos. Interestingly, DJ-1 expression was increased in brains of zebrafish under conditions of oxidative stress, indicating that DJ-1 is a part of stress-responsive machinery. Since oxidative stress is one of the major contributors to the development of Alzheimer's disease (AD), we also examined DJ-1 expression in AD brains. Using DJ-1 specific antibodies, we failed to detect a robust staining of DJ-1 in brain tissues from control subjects. However, DJ-1 immunoreactivity was detected in hippocampal pyramidal neurons and astrocytes of AD brains. Therefore, our results strongly suggest that DJ-1 expression is not necessary during zebrafish development but can be induced in zebrafish exposed to oxidative stress and is present in human AD brains.

## Background

Parkinson's disease (PD) and Alzheimer's disease (AD) are the two most common neurodegenerative disorders. PD is characterized by loss of dopaminergic (DA) neurons in the substantia nigra and accumulation of intraneuronal inclusions known as Lewy bodies. AD is characterized by two major hallmarks, neurofibrillary tangles and neuritic plaques. Genetic studies of PD have demonstrated that two autosomal dominant genes (α-synuclein and LRRK2) [1-3] and three autosomal recessive genes (Parkin, PINK1, and DJ-1) are linked to PD [4-6]. Earlier reports indicate that DJ-1 is expressed in astrocytes and neurons in both control and PD brains, and it is not a major component of Lewy bodies, the pathological hallmark of PD [7,8]. Interestingly, DJ-1 has been reported to co-localize with Tau in neurofibrillary tangles from brains of AD patients, suggesting that PD-linked DJ-1 may play a role in AD [8,9]. Recent studies have found that DJ-1 is enriched in brain tissue containing insoluble hyperphosphorylated tau [10]. In addition, accumulation of acidic isoforms of DJ-1 monomers [10] and basic isoforms of DJ-1 dimers [11] have been found in brains of PD and AD brains. These studies have shown that DJ-1 is subjected to cysteine and methionine oxidation, and the oxidative damage to DJ-1 could be associated with both PD and AD [11].

High levels of DJ-1 mRNA have been detected in neuronal and non-neuronal populations of several regions in mouse brain by *in situ *hybridization [12]. A recent study has shown that DJ-1 expression is up-regulated in neuroblastoma cells exposed to rotenone or 6-hydroxydopamine, which leads to the formation of intracellular reactive oxygen species. Treatment of the mouse hippocampal cell line HT22 with H_2_O_2 _significantly increases the immunoreactivity of DJ-1 [13]. This up-regulation of DJ-1 is suppressed when cells are pre-treated with antioxidant [14].

Pharmacological studies have shown that DJ-1 is needed for the protective effects of certain compounds which are known to inhibit the production of reactive oxygen species [15]. DJ-1 prevents oxidative stress-induced cell death of neuroblastomas, dopaminergic cells and primary neuronal cells in the presence of these compounds and knock down (KD) of DJ-1 eliminates the suppressive effect of these compounds [15]. Overexpression of DJ-1 was found to decrease the expression of BAX and inhibit caspase activation, while KD of DJ-1 increased BAX protein levels, caspase-3 activation and UV exposure-induced cell death. DJ-1 has been found to directly interact with p53, and its sumoylated form inhibits p53 transcriptional activity [16,17]. DJ-1 also stabilizes antioxidant transcriptional master regulator Nrf2 [18] and is involved in the pathway that suppresses JNK1 signaling. DJ-1 was shown to directly target MEKK1 and inhibit MEKK1 kinase activity, and KD of DJ-1 or expression of a mutant L166P form rendered cells vulnerable to death upon stress-induced activation of the MEKK1-SEK1-JNK1 signaling pathway [19].

L166P in addition to E64D, M26I, A104T, and D149A is one of known mutations within DJ-1 that has been identified from familial PD cases. These mutant forms of DJ-1 show different levels of structural alteration (except for E64D due to similar residue replacement), which lead to reduced stability of DJ-1 [20,21]. The L166P form of DJ-1 has an extremely short half life, supporting the notion that mutations in DJ-1 represent a loss-of-function mutation [21].

DJ-1 knockout (KO) mice do not differ from wild type mice with respect to morphology or dopaminergic cell loss [22-24], with no dopaminergic neuronal deficit seen even in aged DJ-1 KO mice [25]. However, isolated mitochondria from DJ-1 KO mice show a 2 fold increase in H_2_O_2_, and DJ-1 appeared to function as a peroxiredoxin-like peroxidase in these animals [23]. In zebrafish, DJ-1 is highly conserved [26], and KD of DJ-1 causes extensive apoptosis [27]. In Drosophila, deficiency in the functional DJ-1 orthologous genes DJ-1α and DJ-1β (by KD and introduction of loss-of-function mutation) renders the fly vulnerable to paraquat-induced oxidative stress, motor impairment and decreased life span [28].

Zebrafish are beginning to be used for aging research, e.g., brief exposure of zebrafish embryos with Dichlorodihydrofluorescein diacetate allows repeated measurement of the level of reactive oxygen species generation in live fish [29]. For neurodegenerative diseases, both zebrafish and goldfish have been used to model the Parkinson's disease [30,31]. Loss of postural reflexes and initiation of movement have been replicated in part in zebrafish and goldfish treated by 1-methyl-4-phenyl-1,2,3,6-tetrahydropyridine (MPTP), a known mitochondrial complex I toxin that has been extensively used to kill DA neurons in a variety of animal models for PD [32-34]. In zebrafish and goldfish, DA neurons are found only in forebrains. It has been proposed that some of these DA neurons in fish forebrains are functionally analogous to DA neurons in both mammalian forebrains and midbrains, especially to the mammalian midbrain ventral tegmental and substantia nigra area [35,36]. Therefore, efforts have been undertaken to generate transgenic zebrafish in which the DA-like monoaminergic neurons are labeled by green fluorescent protein, and elimination of DA-like neurons in zebrafish forebrains is expected to mimic the events implicated in human PD pathogenesis [37].

In this report, we compared KD of DJ-1 to oxidative stress in zebrafish embryos. In addition, we examined DJ-1 expression in the adult fish brain by treating live animals with H_2_O_2_. We found that KD of DJ-1 and H_2_O_2 _had similar effects on islet-1 positive neurons (islet-1 is a LIM (Lin11, Isl-1, Mec-3) homeodomain protein), with both groups of embryos showing an increased number of apoptotic cells. Since the oxidative stress is one of the major contributors to the AD pathogenesis [38,39], we evaluated DJ-1 immunoreactivity in brain sections from AD and control cases and found a robust neuronal staining of DJ-1 in post mortem AD brain tissues.

## Methods

### Fish strains

Embryos were obtained from natural spawning of wild-type (Tubingen longfin strain) adults and were raised and staged according to Kimmel et al. [40].

### Antisense morpholino injection

DJ-1 MO, directed against the 5' untranslated region (UTR) of DJ-1 (GeneTools, Corvallis, OR, USA) was injected into fertilized zebrafish eggs at the one-cell stage at which time morpholinos can rapidly spread into all cells. The morpholino sequences were as follows: DJ-1 MO, 5'-GCGTCTAATAACCTGTGCGTGTCTG-3' control MO, 5'-CCTCCTACCTCAGTTACAATTTATA-3'.

The above standard control MO was synthesized against the zebrafish β-globin intron. MO oligonucleotides were dissolved in 1× Danieu solution at 5 mM, diluted to 0.1 mM prior to injection, and ~4 ng was injected into each embryo. After injection, embryos were incubated at 28.5°C in embryo medium [41] containing 0.002% 1-phenyl-2 thiourea to inhibit pigment formation until they were de-chorionated, deyolked and lysed in sample buffer at 48 hours post fertilization (hpf) for western blot, or fixed overnight in 4% paraformaldehyde.

### TUNEL staining

TUNEL assay was performed using the TMR-RED in situ cell death detection kit [42] according to the manufacturer's instructions. Optical sections were taken with a Zeiss LMR510 confocal Microscope.

### *In situ *hybridization and immunohistochemistry

In situ hybridization was carried out according to standard protocols [43] using a probe against zebrafish tyrosine hydroxylase (TH). Single-stranded RNA probes against TH from the cDNA clone in the pBluescript II KS+ vector (from Dr. Su Guo, University of California at San Francisco, CA) was synthesized using T7 RNA polymerase after linearization by restriction digest and then labeled with digoxigenin-UTP (Roche, Basel, Switzerland). For immunohistochemistry, standard protocols were followed using 1% Triton X-100 in phosphate-buffered saline to permeabilize the embryos and Vector VIP peroxidase substrate kit for the colorimetric reaction (Vector Laboratories, Burlingame, CA, USA). Over one dozen embryos were examined for each experiment. The antibody 39.4D5 against islet-1 was obtained from the Developmental Studies Hybridoma Bank maintained by The University of Iowa.

Human postmortem cortical tissue was collected in accordance with Institutional Review Board-approved guidelines at Brigham and Women's hospital. Blocks of cortex were fixed for ~2 hrs in 10% neutral buffered formalin, as previously described [44]. Paraffin-embedded 8 μm serial sections were blocked for 1 hr in 10% goat serum (for polyclonal DJ-1-N), 10% rabbit serum (for polyclonal Park7), or 10% horse serum (for monoclonal KAM-SA100 or E2.19) and incubated overnight at 4°C with primary antibodies DJ-1-N (1:50), Park7 (1:100, against C-terminus of DJ-1, from Abcam), KAM-SA100 (1:100, against full length DJ-1, from Stressgen), E2.19 (1:100, against full length DJ-1, from Dr. Y. Hod and Abcam)[45]. For preabsorption, 5 μg of DJ-1 peptide per μl of DJ-1 serum was used. For double immunofluorescence, rabbit polyclonal anti-GFAP (1:500, Dako) and DJ-1-N (1:50) were used.

### Western blotting

Human, mouse and zebrafish brain samples were homogenized in lysis buffer containing 0.2% NP-40. The lysates were collected after 1 hr of centrifugation at 100 000 × g (human and mouse) or after 5 min of centrifuge at 18,000 × g (zebrafish). Individual de-chorionated and de-yolked embryos were lysed, and all samples were heated in 1 × sample buffer (3% sodium dodecyl sulfate, 3% β-mercaptoethanol, 15% glycerol, and bromophenol blue) and then separated by sodium dodecyl sulfate polyacrylamide gel electrophoresis. Antibodies KAM-SA100 (1:1000, Stressgen) and anti-DJ-1-N (1:500) and an enhanced chemiluminescence system (Amersham, Piscataway, NJ, USA) were used to detect the western blots.

### Exposure of Aβ in cultured cells

Wildtype CHO cells and APP stably transfected CHO cells (CHO+APP) were grown for 24 hrs before the conditioned media were collected for the measurement of Aβ levels by ELISA, as previously described [46]. The same conditioned media were applied to PC-12 cells for 24 hr, and cells were collected for the quantification of DJ-1 by Western blot using antibody DJ-1-N.

## Results

### Detection of human and zebrafish DJ-1

**Figure 1:**
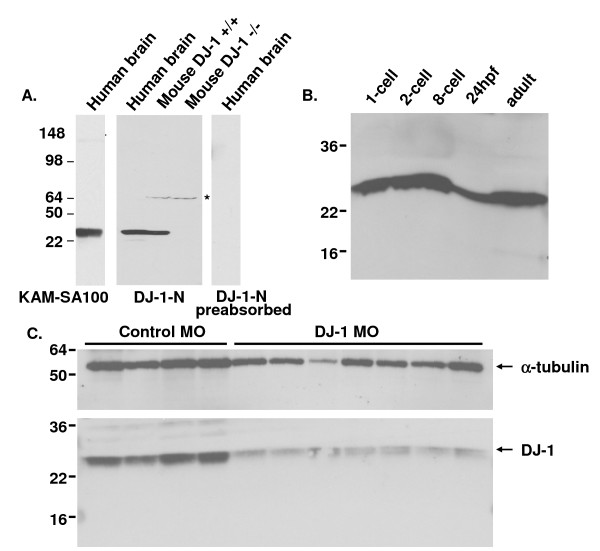
**Knockdown of DJ-1 in zebrafish embryos**. A. Abundant DJ-1 expression in human brain lysate from a control case, as detected by Western Blot (WB) with KAM-SA100 and anti-DJ-1-N antibodies, but not when anti-DJ-1-N was pre-absorbed with the synthetic DJ-1 peptide. Specificity of anti-DJ-1-N antibody was confirmed by detecting DJ-1 in wild type mouse brain lysate but not in the DJ-1 knockout mouse brain lysate. A minor cross-reacting band at ~70 kDa was detected in extracts of both wild type and DJ-1 knockout mouse brain, but not in human brain extracts. B. Zebrafish embryos at different developmental stages and brains of adult zebrafish were lysed for Western blotting with antibody E2.19. High levels of DJ-1 protein were found in embryos at all developmental stages and in adult brains. C. Extracts taken from individual embryos at 24 hpf were run on WB with antibody E2.19 (bottom panel). The same blot was re-probed with antibody against α-tubulin (top panel). DJ-1 protein levels were dramatically reduced in DJ-1 KD embryos compared to control embryos.

We generated an affinity purified polyclonal antibody DJ-1-N against the N-terminus of DJ-1 that recognizes a single band at 23 kDa corresponding to endogenous DJ-1 from human cortex homogenate; this band was absent when the antibody was preabsorbed with its cognate peptide (Fig. 1A). The same band was recognized by a commercially available monoclonal antibody (KAM-SA100) raised against full-length human recombinant DJ-1 (Fig. 1A). We further tested the specificity of this antibody by Western blotting of brain homogenate from a wild type or DJ-1 knockout mouse. Using the antibody DJ-1-N we detected the corresponding 23 kDa band in wild type but not DJ-1 knockout mouse brain lysates (Fig. 1A). We then prepared zebrafish embryos at different developmental stages as well as brain lysates from adult zebrafish for Western blotting using another commercially available monoclonal antibody E2.19 (Fig. 1B). Staining of zebrafish lysates with DJ-1-N showed the same results (data not shown). We found that DJ-1 was expressed early in embryonic development and persisted into adulthood (Fig. 1B).

### Knockdown of DJ-1 in zebrafish embryos

Using the human DJ-1 sequence, we identified multiple zebrafish ESTs and obtained the complete zebrafish DJ-1 sequence by the alignment of these ESTs with genomic zebrafish DJ-1 sequences from the Sanger Institute. We only found one DJ-1 homologue in zebrafish. Zebrafish DJ-1 has 4 exons that encode 189 amino acids, and the protein is 87% homologous to human DJ-1. cDNA from zebrafish embryos at the 1-somite stage was prepared and the full-length zebrafish DJ-1 cloned. The sequence of cDNA was confirmed and found to be identical to the one previously reported [27].

A morphant technology that uses morpholino phosphodiamidate anti-sense oligonucleotides (morpholinos, MO) to target genes in vivo [47-49] was used to knock down DJ-1. We used a stock concentration of 0.5 mM MO targeting DJ-1 and injected DJ-1 MO into embryos at the 1-cell stage. In all experiments, DJ-1 MO injected embryos were compared to embryos injected with a standard control MO targeting the β-globin intron. To confirm the reduction of DJ-1 protein, we examined protein extracts from these embryos at 48 hours post fertilization (hpf) on Western blots (Fig. 1C). DJ-1 was similarly reduced in individual embryos injected with DJ-1 MO compared to control MO injected embryos. The same blot was probed with antibody against α-tubulin as a loading control. Since we transiently knocked down DJ-1 expression, it was different from DJ-1 null animal, as we failed to see a lasting effect of DJ-1 morpholino at 72 hpf (data not shown). In contrast to DJ-1 null mouse which was used for our antibody characterization (Fig. 1A), a lack of DJ-1 null zebrafish prevents us from analyzing animals at later development and adult stages. However, although DJ-1 MO did not completely eliminate DJ-1, the reduction of DJ-1 protein was prominent and consistent among all injected embryos at 48 hpf, the time point when we performed all analyses.

### DJ-1 expression is induced in zebrafish under oxidative stress and retains cell survival

**Figure 2:**
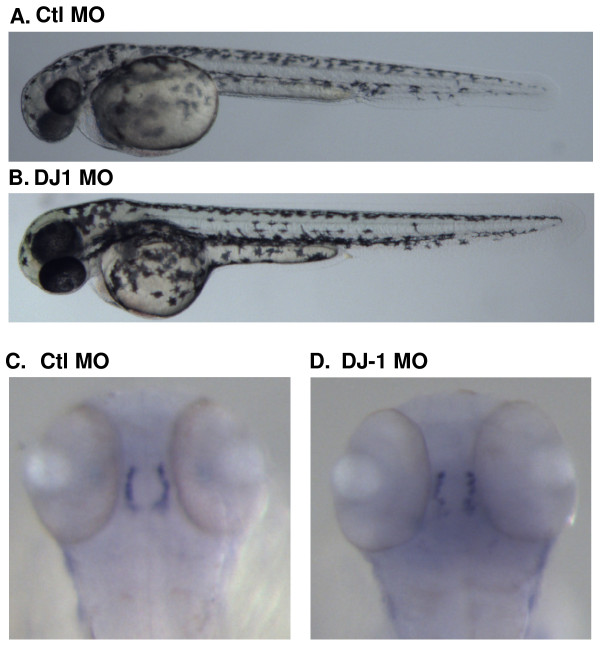
**DJ-1 expression is not essential for proper zebrafish development**. A, B. Embryos were injected with control or DJ-1 MO at the one cell stage, and images were acquired at 48 hpf. DJ-1 KD fish did not differ in morphologic phenotype compared to control MO injected zebrafish. C, D. Embryos injected with control or DJ-1 MO were fixed at 48 hpf, followed by in situ hybridization using a probe against TH. TH staining of control or DJ-1 MO injected embryos was almost identical.

Both control and DJ-1 MO injected embryos shared an almost identical morphology (Fig. 2A), and no dramatic change in phenotype was observed in DJ-1 MO injected embryos (Fig. 2B). Since a loss of DJ-1 in humans leads to PD that is characterized in part by a gradual loss of tyrosine hydroxylase (TH)-positive dopaminergic neurons, we examined the expression of TH in zebrafish embryos by in situ hybridization. We failed to observe any alteration in TH staining in the DJ-1 KD embryos compared with control MO injected embryos at 48 hpf (Fig. 2C, D) suggesting that formation of dopaminergic like neurons in zebrafish was not affected by a loss of DJ-1 protein.

**Figure 3:**
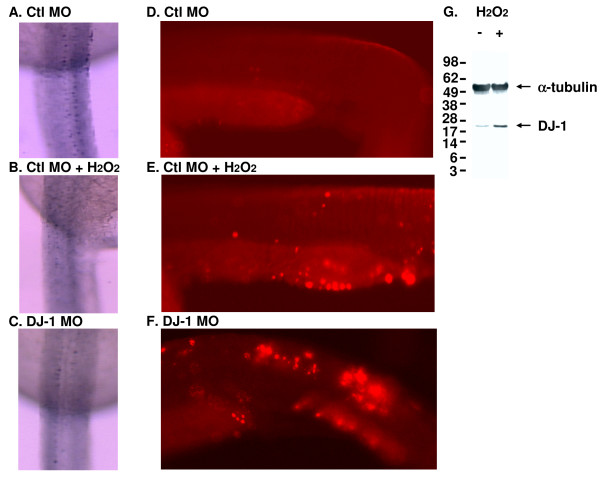
**DJ-1 expression is induced in zebrafish treated with H_2_O_2 _and knockdown of DJ-1 increases the number of apoptotic cells**. A. Control MO injected embryos showed normal distribution of islet-1 positive neurons along the spinal cord, as illustrated by the dorsal view of the spinal cord, anterior to the top of the image. B. Control MO injected embryos treated with H_2_O_2 _showed a slight reduction in islet-1 staining. C. DJ-1 MO injected embryos displayed even weaker islet-1 staining. D-F. Control MO (D, E) or DJ-1 MO (F) was injected into embryos at one cell stage; after 24 hr, control MO injected embryos were treated with 0.03% H_2_O_2 _for 30 min. Compared to control MO injected embryos (D), there was an increase of TUNEL positive cells in the tails of H_2_O_2 _treated embryos (E) and DJ-1 MO injected embryos (F). The lateral view of the trunk region of zebrafish was illustrated with anterior to the left of the image. The number of apoptotic cells in the tails of H_2_O_2 _treated embryos (E) and DJ-1 KD embryos (F) is higher than that in control MO injected embryos (D). G. Adult zebrafish were treated with 0.03% H_2_O_2 _for 30 min before brains were harvested and lysed for Western blot. α-Tubulin (an internal sample loading control) was detected over the upper portion of the blot, and the bottom portion of the blot was detected with antibody DJ-1-N. While the levels of α-tubulin were not changed in the presence of H_2_O_2_, the levels of DJ-1 were increased.

We further examined islet-1 positive primary neurons that play an important role during zebrafish neural development. After control and MO injected embryos were fixed at 48 hpf, we carried out immunostaining using an antibody against islet-1, as we have previously reported [50]. We also treated control embryos with 0.03% H_2_O_2 _to induce an environment of oxidative stress. We found that control MO injected embryos showed well defined islet-1 positive neurons along the spinal cord (Fig. 3A). In the presence of H_2_O_2_, staining in control DJ-1 injected embryos was slightly reduced, but islet-1 positive cells could still be identified (Fig. 3B). Similarly, DJ-1 KD embryos showed weak staining of islet-1 positive cells (Fig. 3C).

When the same set of embryos was examined for the presence of apoptotic cells by TUNEL staining, control MO injected embryos yielded fewer apoptotic cells (Fig. 3D), but the treatment of these embryos with H_2_O_2 _increased the number of apoptotic cells (Fig. 3E). A similar result was obtained in DJ-1 KD embryos, which showed increased TUNEL staining, i.e., an increased number of apoptotic cells (Fig. 3F).

Since we found that DJ-1 was expressed throughout development and into adulthood (Fig. 1B), we explored the regulation of DJ-1 expression in adult fish under a stressful condition, such as oxidative stress. We created an environment of oxidative stress by exposing adult fish to 0.03% H_2_O_2 _in their tank water. We found increased levels of DJ-1, by western blot, in the brains of fish exposed to 0.03% H_2_O_2 _compared with fish from the control environment (Fig. 3G).

### DJ-1 is expressed in pyramidal neurons in AD hippocampus

**Figure 4:**
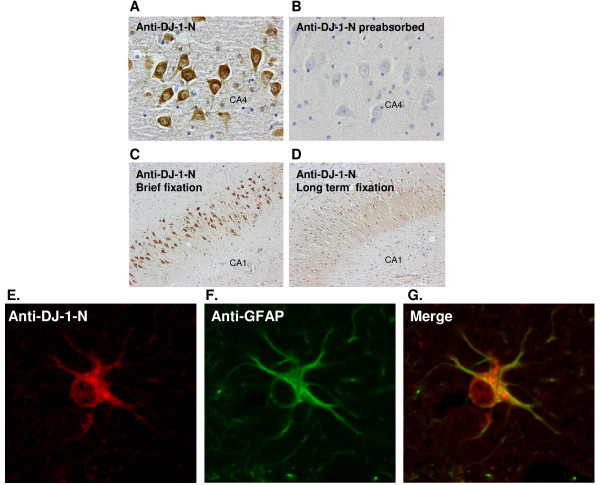
**DJ-1 is detected in neurons from briefly fixed post mortem AD brains**. A. Affinity purified antibody DJ-1-N detected neurons in brain section from AD cases. Staining was observed in pyramidal neurons of CA1, CA2 and CA3 subregions. B. Absence of staining in AD brain with anti-DJ-1-N preabsorbed with DJ-1 peptide, compared to an adjacent section immunostained with anti-DJ-N (A). C. DJ-1 immunoreactivity in a briefly fixed (~2 hours) section was easily detected. D. DJ-1-N failed to stain a routinely fixed (~2–4 weeks) section from the same AD case. E-G, Co-Staining of DJ-1 and GFAP in astrocytes of human brain sections. A routinely fixed section from control brain was stained with antibody DJ-1-N (E) and GFAP (F). Double immunofluorescent overlay indicates the location of DJ-1 in astrocytes (G). Magnification: A, B = 40×; C, D = 20×.

Because oxidative stress is one of the major contributors to AD [38,39], we examined whether up-regulation of DJ-1 occurred in human brains undergoing a neurodegenerative process. We analyzed brain tissue from individuals with well-defined cases of AD. DJ-1 was examined in briefly fixed hippocampal sections from postmortem AD brains using our newly generated, affinity purified DJ-1-N antibody (Fig. 4A) (n = 10, age range: 77–92, Braak Stage: III-VI) [51] and non-demented control cases (n = 9, age range; 52–91). The intensity of the staining was variable among the AD cases, although it did not seem to correlate with the severity of the disease based on Braak staging [51] (data not shown). There was an absence of staining when the DJ-1-N antibody was preabsorbed with synthetic DJ-1 peptide prior to incubation on sections (Fig. 4B) and when primary antibody was omitted (data not shown). Brief fixation (< 2 hours) (Fig. 4C), as opposed to long-term fixation (2–4 weeks) (Fig. 4D) was critical for optimal neuronal DJ-1 immunostaining. We also explored DJ-1 expression in astrocytes. Double immunofluorescence labeling with anti-DJ-1-N (Fig. 4E) and anti-GFAP antibody (Fig. 4F) further confirmed the presence of DJ-1 in astrocytes (Fig. 4G).

**Figure 5:**
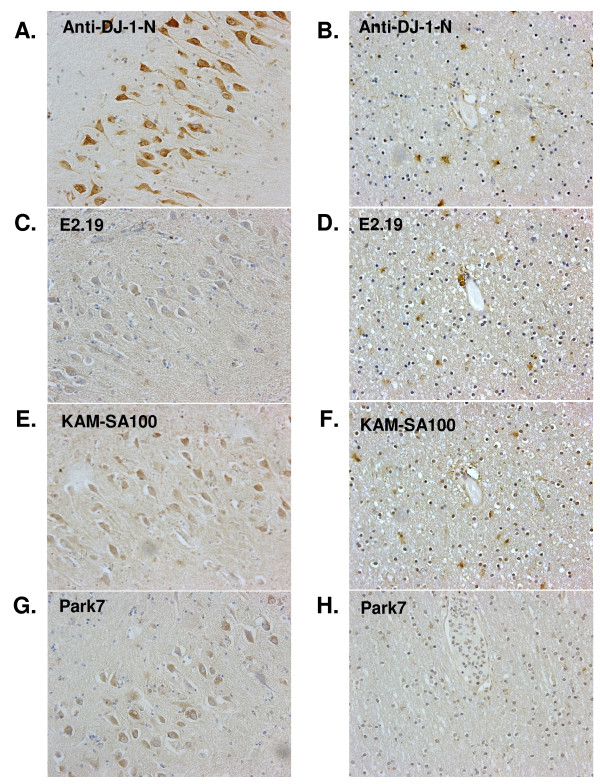
**DJ-1 is detected in both neurons and astrocytes by multiple antibodies**. Antibody DJ-1-N (A) has stronger neuronal staining than three commercially available DJ-1 antibodies, E2.19 (C), KAM-SA100 (E), and Park7 (G) that also detected DJ-1 in neurons in all adjacent briefly fixed sections of the same AD patient. DJ-1-N (B), E2.19 (D), and KAM-SA100 (F) detected immunoreactivity in astrocytes in routinely fixed sections, and Park7 showed a much weaker staining of astrocytes (H). Magnification: 20×.

Similar to DJ-1-N (Fig. 5A), three commercially available DJ-1 antibodies, E2.19 (Fig. 5C), KAM-SA100 (Fig. 5E), and Park7 (Fig. 5G) were able to detect DJ-1 in neurons in adjacent sections of the same AD patient, but the staining was much weaker. Anti-DJ-1-N (Fig. 5B), E2.19 (Fig. 5D), and KAM-SA100 (Fig. 5F) detected astocyte DJ-1 in briefly, as well as, routinely fixed sections, with Park 7 showing much less staining (Fig. 5H).

**Figure 6:**
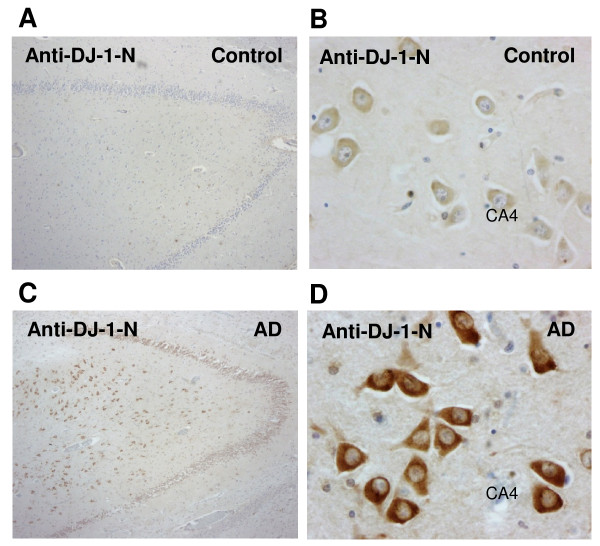
**Enhanced immunostaining of DJ-1 in AD but not control brains**. A, B. control brains (n = 9) showed weak or no DJ-1 staining in pyramidal neurons of the CA1, CA2, CA3 sub-regions and the hilus of the dentate gyrus (CA4). C, D. AD brains (n = 6/10) showed DJ-1 immunoreactivity in pyramidal neurons (cytoplasm and neuronal processes) of CA1–4 regions. Magnification: A, C = 4×; B, D = 40×.

Control brains (n = 9) also showed DJ-1 immunoreactivity in scattered astrocytes (data not shown), but they showed weak or no DJ-1 staining in pyramidal neurons of the CA1, CA2, CA3 sub-regions and the hilus of the dentate gyrus (CA4) (Fig. 6A, B), except for one case (C2, Table 1). In AD brains, 6 out of 10 cases showed moderate to abundant staining of DJ-1 (Table 1). DJ-1 immunoreactivity was observed in pyramidal neurons (cytoplasm and neuronal processes) of CA1–4 regions (Fig. 6C, D). Examination of other cortical areas revealed DJ-1 immunopositive neurons in the enthorinal cortex, frontal lobe and occipital lobe (data not shown). The staining for DJ-1 in brain sections from control and AD subjects was summarized in Table 1.

**Table 1:** Summary of DJ-1 Staining in Brains from Control and AD Subjects

**Subject**	**sex**	**age**	**Diagnosis**	**DJ-1**
C1	F	76	control	-

C2	M	52	control	++

C3	M	53	control	-

C4	F	72	control	-

C5	M	70	control	-

C6	F	60	control	-

C7	M	64	control	-

C8	M	73	control	-

C9	M	91	control	-
A1	M	77	AD	-

A2	F	91	AD	-

A3	F	81	AD	++

A4	F	88	AD	+

A5	M	78	AD	++

A6	M	78	AD	++++

A7	F	92	AD	++

A8	F	84	AD	++

A9	F	86	AD	-

A10	F	80	AD	++

Note: -, none; +, low; ++, moderate; +++, high; ++++, abundant.

**Figure 7:**
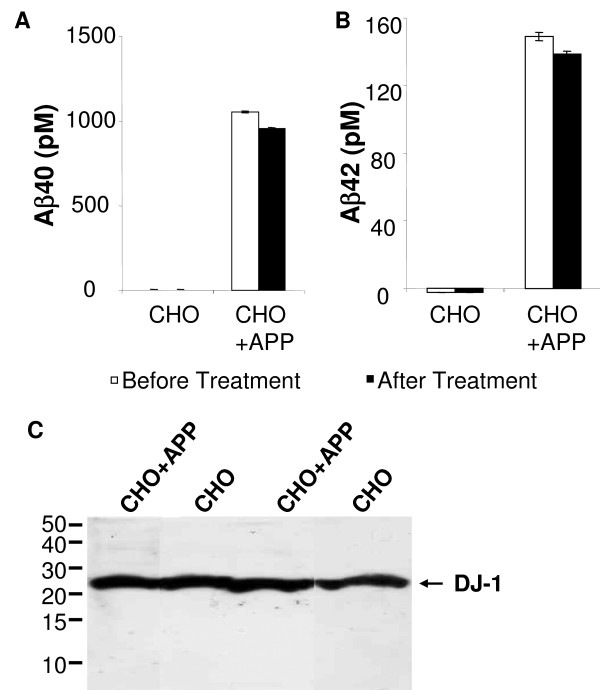
**Lack of changes in DJ-1 expression in the presenceof Aβ**. Conditioned media from CHO and CHO+APP cells were collected for the measurement of Aβ levels and treatment of PC-12 cells. A. High levels of Aβ40 were found in the media from CHO+APP cells before (white bar) and after (black bar) the treatment of PC-12 cells (the standard error of means was illustrated). B. High levels of Aβ42 were found in the media from CHO+APP cells before (white) and after (black) the treatment of PC-12 cells, compared to undetectable amount of Aβ in the media from CHO cells. C. Conditioned media from CHO or CHO+APP cells were applied to PC-12 cells for 24 hr, and cells were collected for the quantification of DJ-1 by Western blot using antibody DJ-1-N. Cells from two independent experiments were collected, and the expression levels of DJ-1 in both sets of PC-12 cells maintained at similar levels.

To search for any mechanistic cause for DJ-1 expression in AD brains, we examined the effect of Aβ on the regulation of DJ-1 expression. We have used two cell lines, CHO that expresses endogenous levels of APP, and CHO+APP that expresses high levels of APP and generates a large amount of Aβ. When we measured the conditioned media from CHO and CHO+APP cells, we found elevated levels of Aβ40 (Fig. 7A, before treatment) and Aβ42 (Fig. 7B, before treatment) in the media from CHO+APP cells, compared to undetectable level of Aβ in the media from CHO cells (Fig. 7A and 7B). We applied conditioned media to PC-12 cells for 24 hrs, and collected media and cells for the quantification of Aβ and DJ-1. We found that the conditioned media continued to carry high levels of Aβ40 (Fig. 7A, after treatment) and Aβ42 (Fig. 7B, after treatment). Despite of the Aβ exposure, the expression levels of DJ-1 in PC-12 cells maintained at similar levels (Fig. 7C). Therefore, endogenous DJ-1 expression in PC-12 cells did not change in the presence of extracellular Aβ.

## Discussion and conclusion

It is known that oxidative stress and mitochondrial dysfunction contribute to the pathogenesis of AD and PD. In AD, Aβ may directly interact with mitochondria and lead to increased free radical production; in PD, DJ-1, parkin and PINK1 are all linked to oxidative stress and/or mitochondrial dysfunction {Reviewed by [52,53]}. In this study, we aimed to examine the relationship between DJ-1 and oxidative stress in zebrafish and also explore the spatial association of DJ-1 to neurons in AD brains.

Consistent with earlier studies in DJ-1 KO mice [22-24], KD of DJ-1 in zebrafish embryos did not result in a change in TH expression {[27] and this study}. Previous studies have focused on pharmacologic treatment of zebrafish to reduce the number of DA-like neurons in zebrafish, e.g., MPTP, rotenone, and paraquat [34]. Extensive loss of DA neurons found in human forebrains and midbrains are responsible for most symptoms that are observed in PD patients, as these DA neurons control postural reflexes and initiation of movement. Genetic ablation of DA neurons becomes possible in light of a successful creation of a transgenic zebrafish expressing GFP in DA-like monoaminergic neurons [37]. Results from our study suggest that knocking down DJ-1 alone is not sufficient to reduce TH-positive DA-like neurons in zebrafish. Instead, a combination of DJ-1 knockdown/knockout and neuronal insult is needed to model pathogenesis in brains of zebrafish. Comparing embryos injected with control MO and those injected with DJ-1 MO, a slightly reduced staining of islet-1 positive neurons was observed in the latter group. Islet-1 expresses at the earliest stage of neural differentiation. It is highly conserved during evolution, and is expressed in the many functional classes of primary neurons at 24 hpf [54-58]. The decrease in the number of islet-1 positive cells in the DJ-1 KD embryos was not high enough to cause abnormal neuronal development, with the DJ-1 MO injected embryos showing hardly any morphologic difference compared to the control MO injected embryos {[27] and this study}.

We found that DJ-1 KD embryos have an increased number of apoptotic cells, consistent with an earlier report showing a p53 dependent apoptosis in DJ-1 KD zebrafish embryos [27]. In cultured cells, DJ-1 directly interacts with p53 to inhibit its transcriptional activity [16]. Since knocking down DJ-1 increases BAX protein levels and caspase-3 activation in cultured cells, knocking down DJ-1 in zebrafish likely undergoes similar pathways that lead to enhanced apoptosis. Another pathway that may be activated in the absence of DJ-1 is the MEKK1-SEK1-JNK1 signaling pathway, as DJ-1 has been shown to directly target MEKK1 and inhibit MEKK1 kinase activity [19]. Knocking down DJ-1 may trigger this pathway thereby increasing the number of apoptotic cells.

Recent studies have shown that DJ-1 expression is up-regulated in cultured cells in an environment that promotes the formation of intracellular reactive oxygen species [13,14]. We have studied the effects of high levels of H_2_O_2 _on DJ-1 expression in the brains of adult zebrafish in vivo. For the first time, we found an increase in DJ-1 protein in brains of adult fish subjected to a stressful environment such as oxidative stress. While it is difficult to discern whether the increase in DJ-1 was derived from neuronal or glial sources, we predict that it was a global response from exposure to H_2_O_2 _in the environment.

To further address this relationship, we utilized brain sections from post mortem AD and control subjects. We found that the avidity of different antibodies and the methods for preserving brain tissues were critical for revealing DJ-1 immunostaining. Whereas antibodies raised against the C-terminus of DJ-1 (Park7) [8,9] or full-length DJ-1 (KAM-SA100 and E2.19) [7,8] predominantly detect reactive astrocytes with less robust neuronal staining, our N-terminal DJ-1-N antibody efficiently stained both astrocytes and pyramidal neurons. The specificity of our affinity purified DJ-1-N was demonstrated by our comparison of tissue lysates from wild type and DJ-1 KO mouse brains. Furthermore, DJ-1-N only detected a single band on our Western blot of human brain lysates. Therefore, immunostaining of human brain sections with DJ-1-N was highly specific, and the robust signals from AD brain sections represent high levels of DJ-1 protein in these neurons.

Our comparison staining with DJ-1-N and other DJ-1 antibodies (E2.19, KAM-SA100 and Park7) reveals an important finding on the potential binding region of DJ-1 when it dimerizes. Earlier reports, including ours, have demonstrated that DJ-1 dimerizes and forms a high molecular weight complex in cultured cells and human brains [21,59]. It seems that the mid- and C-terminal regions of DJ-1 could be involved in dimerization and are no longer accessible by antibodies that recognize these regions. However, the N-terminal region of DJ-1 may still be exposed and not involved in binding in the dimer or high molecular weight complex. DJ-1-N may therefore efficiently detect the exposed antigen in brain sections. This finding is identical to what we have found in Aβ dimers, where N-terminal antibodies more effectively capture dimeric Aβ compared to antibodies recognizing mid- and C-terminal regions of Aβ [46]. Whether the N-terminus of DJ-1 is somehow altered during long term fixation is not clear, as strong staining of DJ-1 only in briefly fixed brain sections suggests that the method of preserving post mortem brains plays an important role.

The intriguing finding of positive staining of DJ-1 in AD brains but not control brains has important implications for both PD and AD pathogenesis. In brains of AD patients, the neuritic plaque composed of Aβ is one of the major characteristics of AD pathology. A lack of change in DJ-1 expression in cultured cells exposed to high levels of Aβ suggests that Aβ has no direct effect on steady state levels of DJ-1. During the neurodegenerative process, accumulation of reactive oxygen species renders neurons, such as pyramidal neurons around hippocampal area, extremely vulnerable, and gradual neuronal loss is inevitable when the brain is exposed to insults. The fact that DJ-1 itself is subjected to cysteine and methionine oxidation [11] as well as the increment of DJ-1 expression in zebrafish under oxidative stress provides important clues to our findings in human brains. It seems that the damaged DJ-1 failed to prevent oxidative stress-induced gradual neuronal death when neurons in AD brains were exposed to an environment with high levels of reactive oxygen species. In certain areas of the brain in AD patients, levels of functional DJ-1 may be reduced, leading to neuronal death. Under these conditions, human brain may respond with an increased expression of DJ-1, like zebrafish exposed to H_2_O_2_. Therefore, the similarity of increased DJ-1 staining in H_2_O_2 _treated zebrafish and AD brains suggest that a common pathway might be elicited under damaging conditions. As previously reported, DJ-1 is needed to avoid oxidative stress-induced cell death in cultured neuroblastomas, dopaminergic cells and primary neuronal cells [60]. Therefore, our results suggest that surviving neurons found in AD hippocampus expressed high levels of DJ-1 in response to a damaging environment, whereas in control brains few exogenous factors induced the expression of DJ-1.

Furthermore, DJ-1 is a non-traditional peroxiredoxin-like peroxidase [23], and enhanced levels of DJ-1 in pyramidal neurons in AD brain may simply represent a response to an accumulation of reactive oxygen species in the mitochondria. This is supported by the finding that mitochondria isolated from DJ-1 KO mice have increased levels of H_2_O_2 _[23].

Future studies are necessary to characterize the role of DJ-1 in PD and AD pathogenesis such as whether the DJ-1 protein we found in pyramidal neurons in AD hippocampus is an acidic isoform [10], basic isoform [11], or both. A specific isoform of DJ-1 may lose its protective role in neuronal survival while oxidation of cysteine/methionine in DJ-1 may lead to a detrimental effect on neurons. The novel finding of an increased DJ-1 expression in zebrafish treated with H_2_O_2 _illustrates a new plate form that allows us to examine the role of DJ-1 involved in response to oxidative stress. Investigation of the function/dysfunction of DJ-1 in a whole vertebrate animal exposed to a stressful environment has a direct physiological relevance to human diseases. Therefore, exploring mechanisms of DJ-1 associated with PD and AD pathogenesis will provide insight into common pathways involved in both neurodegenerative diseases. Any newly identified pathways will offer novel drug targets and exciting opportunities for therapeutic intervention.

## Abbreviations

AD: Alzheimer's disease; Aβ: amyloid β-protein; DA: dopaminergic; hpf: hours post fertilization; KD: knock down; KO: knock out; MO: morpholino; MPTP: 1-methyl-4-phenyl-1,2,3,6-tetrahydropyridine; PD: Parkinson's disease; TH: tyrosine hydroxylase.

## Competing interests

The authors declare that they have no competing interests.

## Authors' contributions

SB and JS carried out the biochemical and immunohistochemical analyses, HL carried out zebrafish experiments, TY carried out cell culture experiments, MS, JS, MGS, CAL, and QL participated in the design of the study, SB and WX conceived of the study and draft the manuscript. All authors read and approved the final manuscript.
